# Oral microbiota in preschoolers with rampant caries: a matched case–control study

**DOI:** 10.1007/s00253-024-13362-5

**Published:** 2024-12-11

**Authors:** Yao Wu, Guiding Li, Chang-hai Lyu, Ni Zhou, Hai Ming Wong

**Affiliations:** 1 Department of Pediatric Dentistry, School and Hospital of Stomatology, Kunming Medical University, Yunnan, China; 2 Yunnan Key Laboratory of Stomatology, Kunming Medical University, Kunming, China; 3 Division of Paediatric Dentistry & Orthodontics, Faculty of Dentistry, 2/F Prince Philip Dental Hospital, The University of Hong Kong, Hong Kong SAR, China

**Keywords:** Oral microbiota, Rampant caries, Early childhood, Preschool, Pediatric

## Abstract

**Abstract:**

Rampant caries is identified by rapid onset, severe decay affecting multiple surfaces, and early pulp infection. This case–control study was conducted to investigate the disparities in oral microbiota between children affected by rampant caries and their caries-free counterparts. A total of 88 preschool children, with matched distribution of sex and age in both the case and control groups, participated in this study. Children’s oral health–related behaviors were reported by parents, salivary pH levels were assessed using a portable pen-type pH meter, and supragingival dental plaque was analyzed by 16S rRNA gene sequencing. Children with rampant caries exhibited lower salivary pH levels, poorer toothbrushing habits, and more frequent consumption of sugary snacks. *Veillonella*, enriched in caries-free children, showed a positive correlation with salivary pH levels and a negative correlation with candy consumption. Conversely, *Fusobacterium* and *Neisseria*, more abundant in children with rampant caries, positively correlated with the frequency of candy consumption. Furthermore, *Streptococcus mutans*, *Porphyromonas gingivalis*, and *Bacteroides acidifaciens* were identified as potential oral microbiome markers for differentiating preschoolers with rampant caries from their caries-free peers. *B. acidifaciens*, typically found in the gut, has been rarely reported in the field of oral health. More well-designed cohort studies are recommended to elucidate the mechanisms through which gut microbiota influences rampant caries in pediatric patients and offer insights into effective strategies for caries management in young children.

**Key points:**

*• Lower salivary pH levels in children with rampant caries.*

*• Biomarkers for predicting rampant caries.*

*• Impact of oral health–related behaviors on oral microbiota.*

## Introduction

Rampant caries refers to “a suddenly appearing, widespread, rapid burrowing type of caries, resulting in early involvement of the pulp and affecting those teeth usually regarded as immune to ordinary decay” (Massler 1945). It is well-identified by rapid onset and the presence of severe tooth decay affecting various surfaces of multiple teeth, including surfaces that are typically not susceptible to caries (Winter et al. 1966; Guzmán-Armstrong 2005; Wong 2022). There are three primary types of rampant caries: (i) nursing bottle rampant caries, which are commonly seen in young children who frequently consume milk or other sweetened drinks through bottle feeding; (ii) adolescent rampant caries, typically found in teenagers who have a high intake of sugary foods and beverages; and (iii) xerostomia-induced rampant caries, which occur due to decreased saliva production, leaving the teeth more susceptible to decay (Chandra et al. 2007; Namita 2012; Lestari et al. 2022).

The management of rampant caries poses a multitude of challenges for both dental care providers and patients. The widespread nature of caries in these cases often necessitates extensive and complex dental procedures such as pulp therapy, tooth extraction, and restorative treatments (Vinckier et al. 2001). The situation is further complicated among younger children due to the anxiety that young children may experience, which can lead to poor cooperation during dental treatments. Additionally, patients whose primary dentition was affected by rampant caries are more likely to be affected by rampant caries in their permanent dentition, if comprehensive dental care is not provided (Nagaveni et al. 2019). Rampant caries is associated with salivary pH, inappropriate dietary habit, unfavorable oral hygiene practices, frequent intake of medication containing sugar, radiation exposure to the head and neck, the existence of a systemic ailment, psychological stress, and limited dental services (Namita 2012; Padmanabha et al. 2017; Wong 2022).

Dental caries has a microbiological origin. It is well recognized that acid-producing bacteria, such as *Streptococcus mutans*, are the primary cariogenic bacteria due to their exopolysaccharide production, acid production, and acid resistance (Loesche 1986). However, it is assumed that the occurrence of childhood caries caused by an imbalance in the microecology may be linked to several microorganisms, which is different from the traditional understanding that caries was triggered solely by specific cariogenic bacteria (Zheng et al. 2021). Other bacteria, such as *Lactobacillus*, *Veillonella*, *Actinomyces*, and *Fusobacterium* may also contribute to the occurrence of tooth decay (Simón-Soro et al. 2014; Chumponsuk et al. 2021; Li et al. 2022). As primary teeth are more vulnerable to tooth decay due to ineffective oral hygiene maintenance and frequent sugary snacking intake, caries control among young children is of utmost importance (Srivastava et al. 2024). Investigating the composition of oral microbiota and associated factors among children with and without rampant caries is crucial to exploring optimal interventions for preventing rampant caries. However, the study children recruited in the prior studies were mainly diagnosed with early childhood caries (ECC), rather than rampant caries. ECC refers to the presence of one or more decayed (non-cavitated or cavitated lesions), missing (due to caries), or filled tooth surfaces in a child aged 71 months or younger (Drury et al. 1999). The participants of the previous studies showed less severe caries. The profile of oral microbiota among young children with rampant caries remains unclear.

To address this research gap, we compared the diversity of oral microbiota among preschool children with rampant caries to their caries-free counterparts. Additionally, as multiple factors can contribute to the onset of rampant caries, this study also investigated the impact of salivary pH levels, tooth brushing habits, and dietary habits on the variation of oral microbiota among the recruited children. By delving into these factors, we aimed to identify the dominant bacteria among young children with rampant caries, and to explore the potential factors that contribute to the occurrence of rampant caries among young children.

## Materials and methods

### Study design and participant recruitment

Ethical approval was granted by the local Ethics Committee of the Affiliated Stomatological Hospital of Kunming Medical University, China (KYKQ2021MEC089). This study was reported following the Strengthening the Reporting of Observational Studies in Epidemiology (STROBE) statements (von Elm et al. 2014). Parents or legal guardians of the recruited children signed the consent form prior to the commencement of this study.

This was designed as a matched case–control study. The case group consisted of preschoolers affected by rampant caries (CR group), consecutively recruited from children who were seeking dental care at the Department of Pediatric Dentistry and Preventive Dentistry, the Affiliated Stomatological Hospital of Kunming Medical University, between July 1 and August 31, 2022. The control group consisted of caries-free children (CF group), who were recruited from local kindergartens.

The inclusion criteria for children in the CR group were (i) children registered at the Affiliated Stomatological Hospital of Kunming Medical University; (ii) aged between 2 and 5 years; (iii) diagnosed with rampant caries by pediatric dentists in clinical setting; (iv) no prior dental treatment received. Children who met any of the following items were excluded: (i) received topical fluoride application within the past 3 months; (ii) administration of antibiotics within the past 3 months; (iii) recent intake of medications for severe allergies or undergoing tonsillectomy in the past 6 months; (iv) undergoing orthodontic treatment; (v) presence of systemic diseases, cleft lip, or cleft palate; (vi) presence of intellectual and developmental disabilities. The participants in the CF group had no signs of dental caries. The exclusion criteria for children in the CF group were consistent with those in the CR group. Additionally, to control for confounding factors, children in the CF group were matched for age and sex with those in the CR group.

### Intra-oral examination

Children’s caries status was evaluated by a single calibrated pediatric dentist (WY) at the clinic setting in line with the World Health Organization (WHO, 2013) criteria. The intra-rater reliability was 0.93. The number of decayed, filled, and missing (due to caries) primary tooth surfaces (dmfs) was calculated to evaluate caries status. Children who had 10 or more carious surfaces (dmfs ≥ 10) within 1 year and at least one tooth with early pulp involvement (Chandra et al. 2007; Namita 2012) were assigned to the case group, while those children without caries (dmfs = 0) were assigned to the control group.

### Oral health–related behaviors investigation

The oral health–related behaviors of the recruited children were investigated via a structured questionnaire, which was completed by parents. The following items were covered, namely, frequency of candy consumption (“Never or occasionally” “Sometimes” “Often”), frequency of toothbrushing (“Never or once daily” “Twice daily”), duration of toothbrushing (“Less than 2 min” “2 min or more”), and usage of fluoridated toothpaste (“Yes” or “No”). Two investigators cross-checked the questionnaires to identify any missing items, and missing data were obtained by contacting the parents, either in person or via phone calls. Incomplete questionnaires were excluded from the final analysis.

### Dental plaque collection and salivary pH measurement

Following the intra-oral examination, a sterilized excavator (size #6) was used to collect the supragingival plaque from both the buccal and lingual tooth surfaces of all the primary teeth. The dental plaque was placed in a 2.0-mL cryotube, immediately stored on ice, and subsequently frozen in liquid nitrogen at − 196 °C within 2 h. A disposable pipette was used to collect the non-irritating saliva (1 mL) from the floor of the oral cavity. Prior to salivary collection, the recruited children were instructed to relax for several minutes and sat straight on a chair (Jayaraj and Ganesan 2015). Immediately after collection, a portable pen-type pH meter with flat surface electrode (PH5F, Sanxin, Shanghai, China) was employed to measure the pH values of the salivary samples.

### DNA extraction, amplification, and 16S rRNA gene sequencing

All the plaque samples were transported to the local laboratory in an ice container and subsequently centrifuged at 5000 g for 5 min. The supernatants were then discarded, and the remaining pellets were kept for microbial DNA extraction, using the cetyltrimethylammonium bromide (CTAB) technique. The primers 341F (5′-CCTAYGGGRBGCASCAG-3′) and 806R (5′-GGACTACNNGGGTATCTAAT-3′) were applied to amplify the bacterial 16S rRNA gene V3–V4 region. The polymerase chain reaction (PCR) process was conducted following the method described by Lei et al. (2022). The PCR products were purified with Universal DNA Purification Kit (TianGen, Beijing, China), and were sequenced on the Illumina platforms (NovaSeq 6000, Illumina Inc., CA, USA) at Novogene. The Illumina sequencing raw data has been deposited in SRA database (Ref No. PRJNA1141721).

### Statistical and bioinformatics analysis

The clinical data was analyzed using IBM SPSS Statistics 28.0 (IBM Corp, Armonk, New York). The significance level was set at 0.05. Data entry was crosschecked by two investigators. Regarding the intra-oral examination, the intra-examiner reliability was assessed using Kappa values. The pH levels were recorded as continuous data. As children in the CR and CF groups were matched in terms of age and gender, inter-group comparisons of salivary pH levels were analyzed using paired *t* tests. The chi-squared tests and Fisher’s exact tests were used to compare categorical variables between CR and CF groups when appropriate. Wilcoxon signed-rank test was employed to compare the Chao1 index and Shannon index between the case and control groups. Welch *t* test was employed to analyze the differences in the composition of oral microbiota between children in the CF and CR groups. Paired-end reads were merged using FLASH (V1.2.11), and raw tags were filtered to obtain high-quality Clean Tags (Bokulich et al. 2012; Magoc and Salzberg 2011) . Silva (v138, https://www.arb-silva.de/) was used to classify the bacterial taxa. Operational taxonomic units (OTUs) were assigned to sequences with a similarity of 97% or higher (Gong et al. 2019). The alpha-diversity analysis was conducted by using QIIME 1.9.1 (Park et al. 2022). The estimation of beta diversity was conducted via principal coordinate analysis (PCoA) and non-metric multidimensional scaling (NMDS), based on the unweighted UniFrac distance using R software 4.0.3 with the ade4 package and ggplot2 package (Lu et al. 2023). The analysis of similarities (ANOSIM) was employed to ascertain whether the discrepancies between the groups were markedly greater than the variations within the groups. This is accomplished by evaluating the significance of the differences in UniFrac distance. Linear discriminant analysis (LDA) is a supervised feature extraction machine learning algorithm for dimensionality reduction and classification (Anowar et al. 2021). Linear discriminant analysis effect size (LefSe) was used to identify biomarkers that differentiate between the CR and CF groups. The default LDA score threshold was set to 4, and the species with a *p* value less than 0.05 were selected as species with statistical differences between the two groups. For different classification levels, different numbers of species were selected according to the gradient to construct the random forest model, and a receiver operating characteristic curve (ROC) curve was drawn. Each model was then cross-validated to identify significant species. The correlation between bacterial community and potential factors, such as salivary pH levels, snacking frequency, tooth-brushing frequency, tooth-brushing duration, and usage of fluoride toothpaste, was analyzed by canonical correlation analysis (CCA) as well as the Spearman rank correlation test.

## Results

### Characteristics of the recruited children

Eighty-eight preschool children participated in the present study. Those children were aged between 2 and 5 years, with an average age was 3.14 ± 0.91, and 54.5% were boys. The CR group consisted of 44 preschoolers with rampant caries, and the CF group consisted of 44 preschoolers without any caries. There were no significant differences in family household income or parents’ educational attainment between children in the CR and CF groups. The household income of over 80% of the recruited families was below RMB 20,001. Additionally, among the recruited children, 75% of their parents were both employed full-time. More than half (*n* = 47) of mothers, and nearly 48% (*n* = 42) fathers had completed more than 12 years of education (Table 1).

**Table 1:** Background information of the recruited children (*n* = 88)

	Case group	Control group	Overall
Age	3.14 (0.91)	3.14 (0.91)	3.14 (0.91)
Gender			
Boy	54.5 (24)	54.5 (24)	54.5 (48)
Girl	45.5 (20)	45.5 (20)	45.5 (40)
Father’s education attainment			
≤ 12 years	59.1 (26)	45.5 (20)	52.3 (46)
> 12 years	40.9 (18)	54.5 (24)	47.7 (42)
Mother’s education attainment			
≤ 12 years	52.3 (23)	40.9 (18)	46.6 (41)
> 12 years	47.7 (21)	59.1 (26)	53.4 (47)
Parents employed full-time			
Yes	63.6 (28)	86.4 (38)	75.0 (66)
No	36.4 (16)	13.6 (6)	25.0 (22)
Family household income			
≤ ¥ 10,000	54.6 (24)	29.5 (13)	42.0 (37)
¥ 10,000–20,000	31.8 (14)	52.3 (23)	42.0 (37)
> ¥ 20,000	13.6 (6)	18.2 (8)	16.0 (14)

Age is presented as mean ± SD; the other values are presented as % (*n*)

### Oral examination, salivary pH levels, and oral health–related behaviors

The dmfs scores for children with rampant caries ranged from 11 to 68, with an average score of 29.89 ± 16.06. Significant differences were detected in salivary pH levels (*p* = 0.002), candy-consumption frequency (*p* < 0.001), tooth-brushing frequency (*p* = 0.003), and utilization of fluoride toothpaste (*p* = 0.019). Children without rampant caries exhibited higher salivary pH values than those with rampant caries (7.18 ± 0.28, 6.93 ± 0.42, *p* = 0.002). The proportion of fluoride toothpaste usage was lower among children with rampant caries than among those without caries (36.4% vs. 61.4%, *p* = 0.019). Furthermore, the proportion of twice-daily toothbrushing was higher among those without caries than children with rampant caries (68.2% vs. 36.4%, *p* = 0.003, Table 2).

**Table 2:** Salivary pH and oral health–related behaviors among children in both groups (*n* = 88)

	Case group	Control group	*p* values
Salivary pH	6.93 (0.42)	7.18 (0.28)	0.002
Frequency of candy consumption			< 0.001
Never or occasionally	18.2 (8)	63.6 (28)	
Sometimes	43.2 (19)	31.8 (14)	
Often	38.6 (17)	4.6 (2)	
Frequency of tooth brushing			0.003
Never or once daily	68.2 (30)	36.4 (16)	
Twice daily	31.8 (14)	63.6 (28)	
Duration of tooth brushing			NS
Less than 2 min	77.3 (34)	61.4 (27)	
2 min or more	22.7 (10)	38.6 (17)	
Usage of fluoride toothpaste			0.019
No	63.6 (28)	38.6 (17)	
Yes	36.4 (16)	61.4 (27)	

Salivary pH value is presented as mean ± SD; the other values are presented as % (*n*)

### Sequencing date

**Fig. 1:**
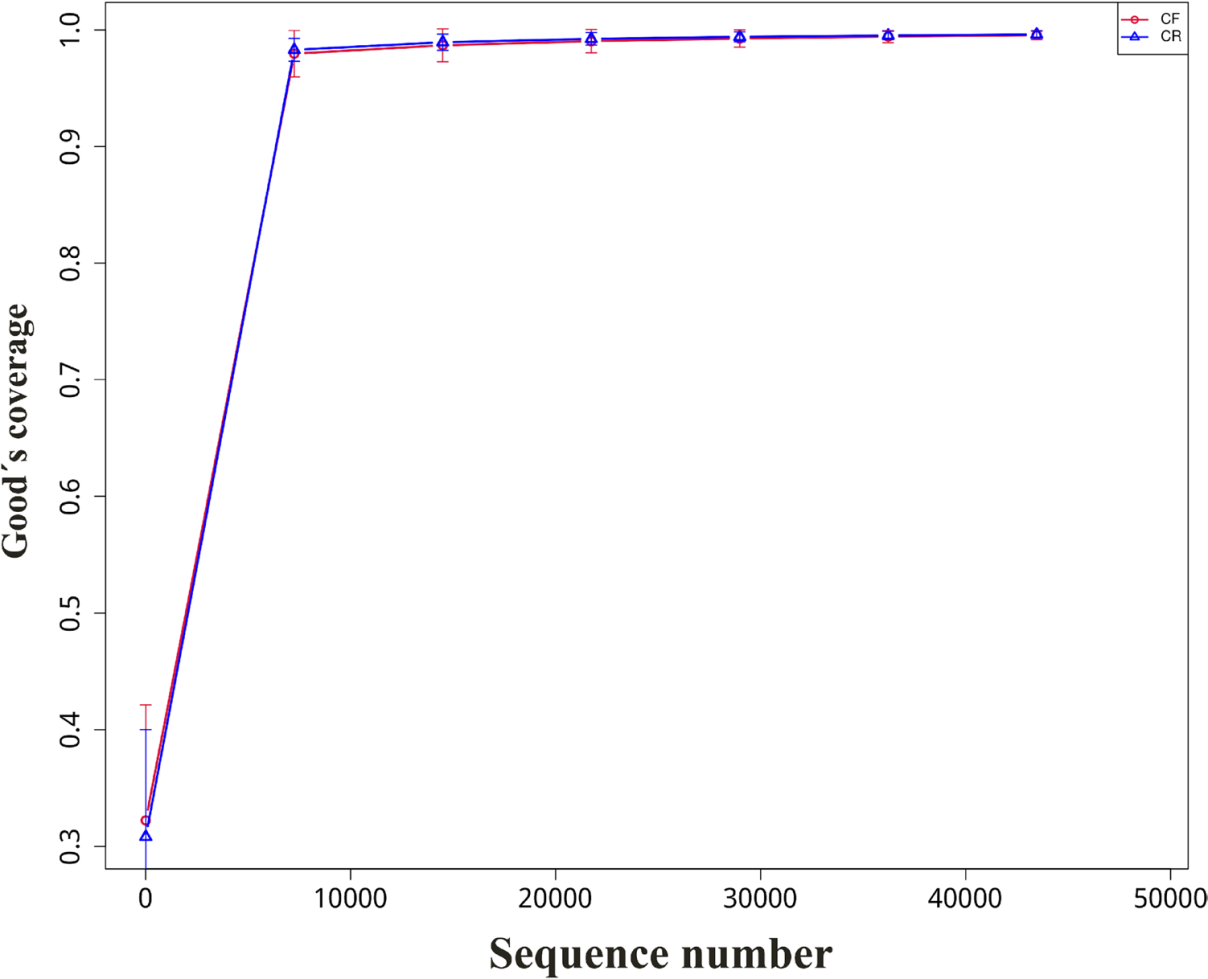
Rarefaction curves for 88 sample collected from caries-free (CF) children and children with rampant caries (CR)

A total of 15,193,162 reads were generated by Illumina sequencing, with an average reads of 72,348 per sample. The final analysis involved 88 plaque samples, including 44 preschoolers with rampant caries and 44 caries-free children. The rarefaction curve depicted the observed alterations in species richness or the quantity of gene sequences in relation to an increasing number of samples. As more samples were included, the rarefaction curve plateau gradually. This observation indicated that the depth and volume of the sequencing data were adequate to generate a reliable depiction of the microbial population present in the plaque samples (Fig. 1).

### Oral microbiota diversity and composition among the recruited children

**Fig. 2:**
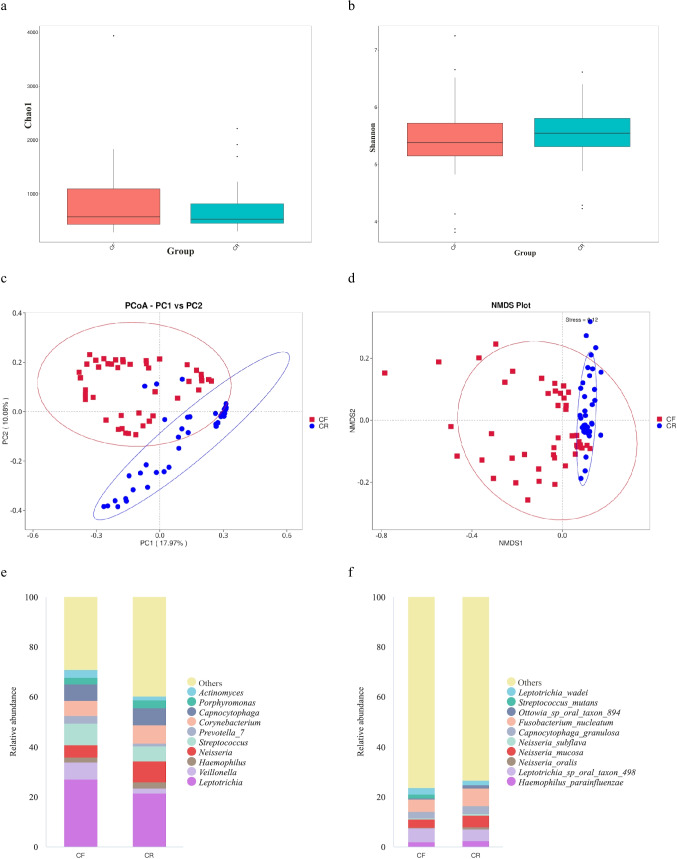
The diversity and oral microbiota composition between caries-free (CF) children and children affected by rampant caries (CR). The alpha diversity is presented in **a** Chao1 index and **b** Shannon index. **c** PCoA (principal coordinates analysis) and **d** NMDS (non-metric multidimensional scaling) are methods used for visualizing high-dimensional data; they indicated significant differences in the microbiome-community structures among children in the CF and CR groups. Distribution of top 10 most abundant **e** genera and **f** species in CR and CF groups. Excluding the unclassified bacteria, *Leptotrichia* was the main genus in the CF and CR groups with a high relative abundance of 27.0% and 21.6%, respectively. *Fusobacterium nucleatum* was the main species in the CR group, while *Leptotrichia* sp. oral taxon 498 was the main species in the CF group

Among preschoolers with rampant caries, the Chao1 index and Shannon index were 760.85 and 0.95, respectively. As to caries-free children, the values of the above indices were 859.38 and 0.94, respectively. No significant differences in Chao1 index nor Shannon index were detected between the case group and the healthy control group (*p* > 0.05, Fig. 2a–b). Based on PCoA analysis (Fig. 2c), if the bacteria in dental plaque were served as the classification standard, there was a partial overlap between rampant caries and caries-free. The NMDS analysis (Fig. 2d) showed that the dental plaque collected from children with rampant caries were clustered. In contrast, the dental plaque samples collected from caries-free children were more dispersed. The results of beta diversity analysis indicated that the CR group was presented with a special and stable bacterial community structure, while the bacterial microbiome structure from the CF group was more diverse.

Regarding the microbial composition among caries-free children, the most abundant genus was *Leptotrichia* (27.0%), followed by *Streptococcus* (8.5%), *Veillonella* (6.9%), *Capnocytophaga* (6.6%), *Corynebacterium* (6.1%), *Neisseria* (4.8%), *Prevotella_7* (3.1%), *Actinomyces* (3.0%), *Porphyromonas* (2.6%), and *Haemophilus* (2.1%). Likewise, *Leptotrichia* (21.6%) was also found to be the most abundant genus among children with rampant caries (Fig. 2e). At the species level, the top 10 dominant species were consistent in both groups, although their proportions varied significantly. *Fusobacterium nucleatum* (7.1%) was the most abundant species detected among preschoolers affected by rampant caries, followed by *Leptotrichia* sp. oral taxon 498 (4.7%), *Neisseria mucosa* (4.5%), *Capnocytophaga granulosa* (3.1%), *Haemophilus parainfluenzae* (2.4%), *Leptotrichia wadei* (1.7%), *Ottowia* sp. oral taxon 894 (1.3%), *Neisseria oralis* (1.0%), *Neisseria subflava* (0.7%), and *S. mutans* (< 0.1%). Additionally, the most abundant species among caries-free children were *Leptotrichia* sp. oral taxon 498 (5.4%, Fig. 2f).

**Fig. 3:**
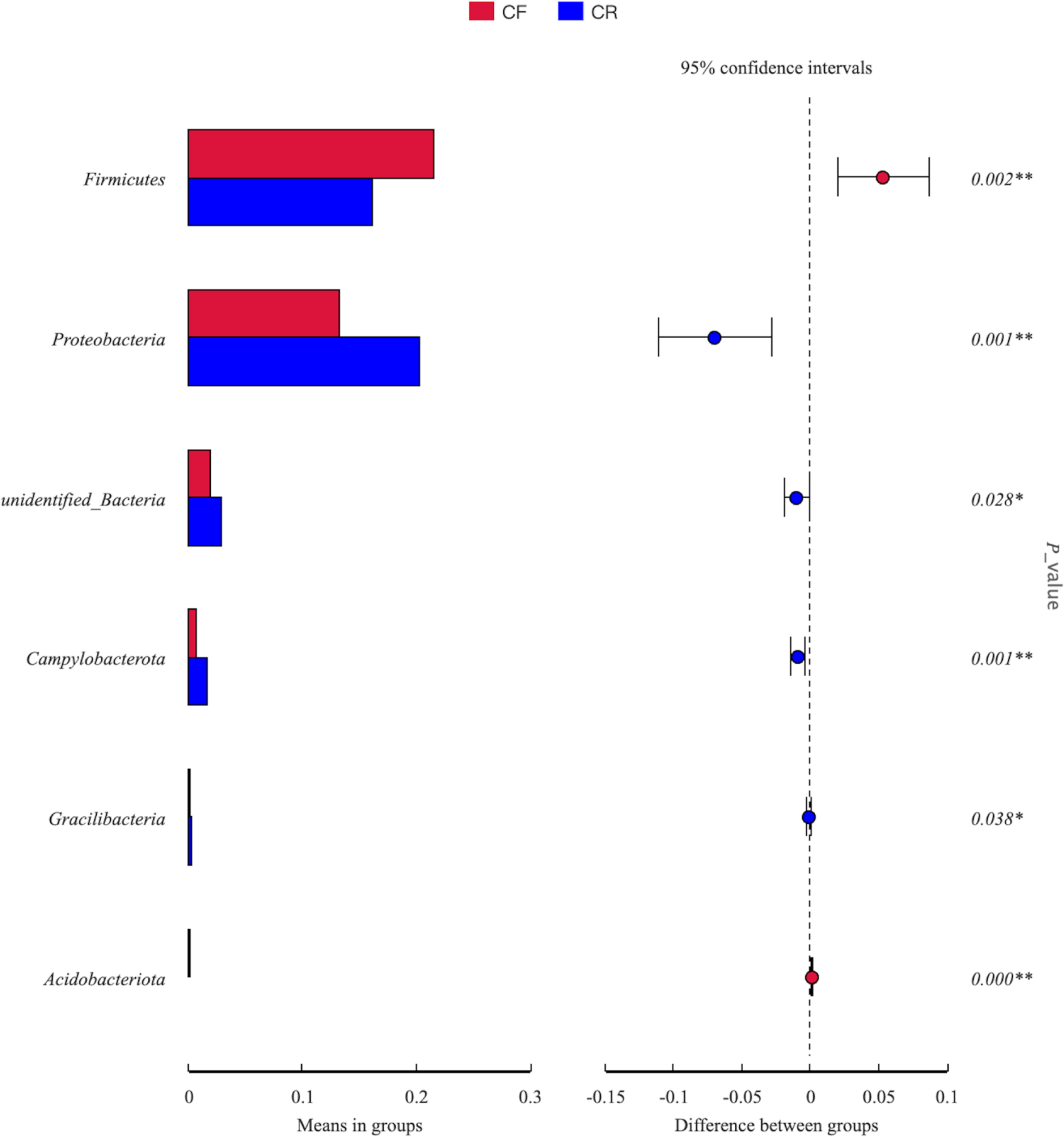
Inter-group comparison of microbial composition at the phylum level between caries-free (CF) children and children affected by rampant caries (CR)

**Fig. 4:**
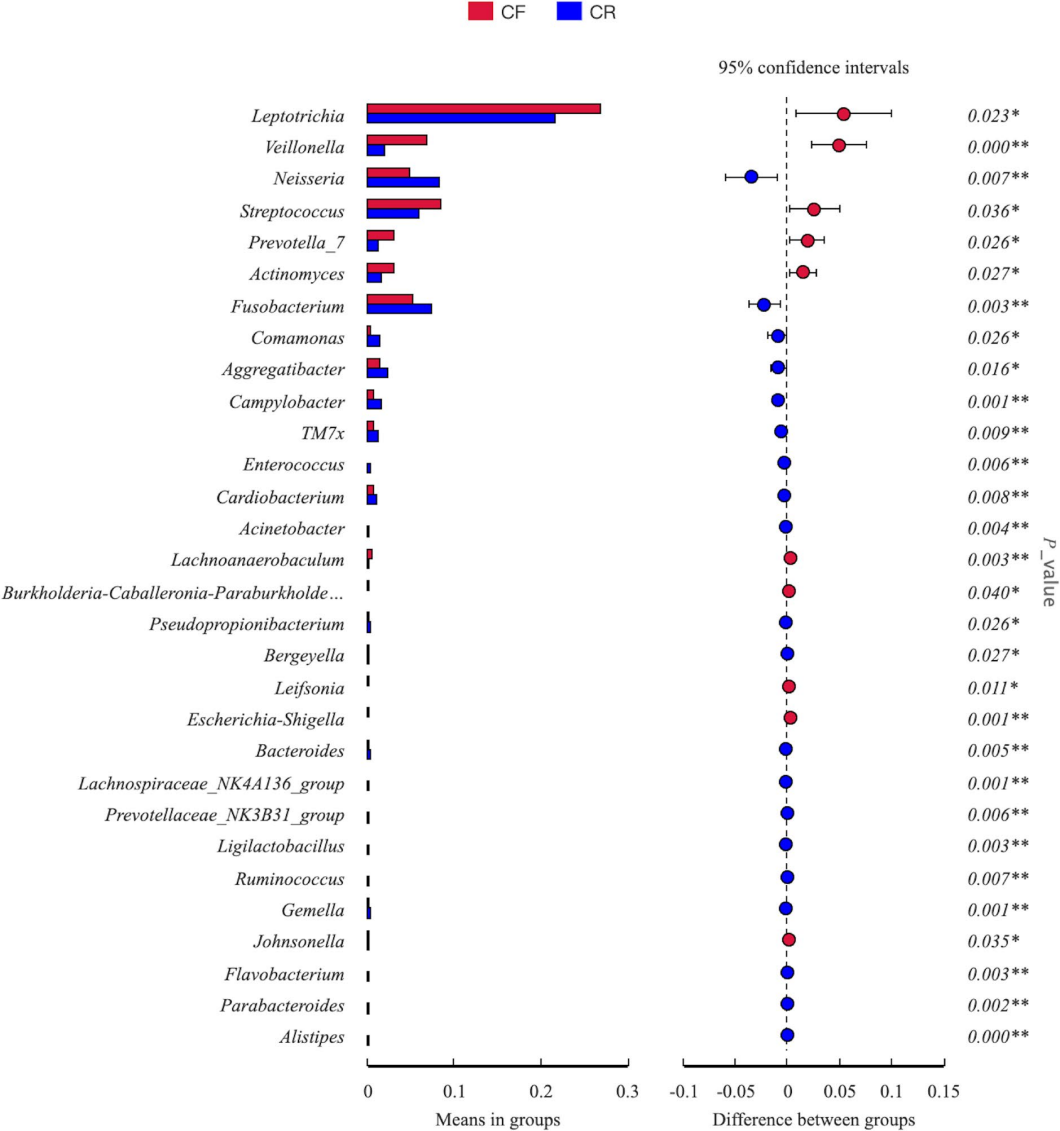
Inter-group comparison of microbial composition at the genus level between caries-free (CF) children and children affected by rampant caries (CR)

**Fig. 5:**
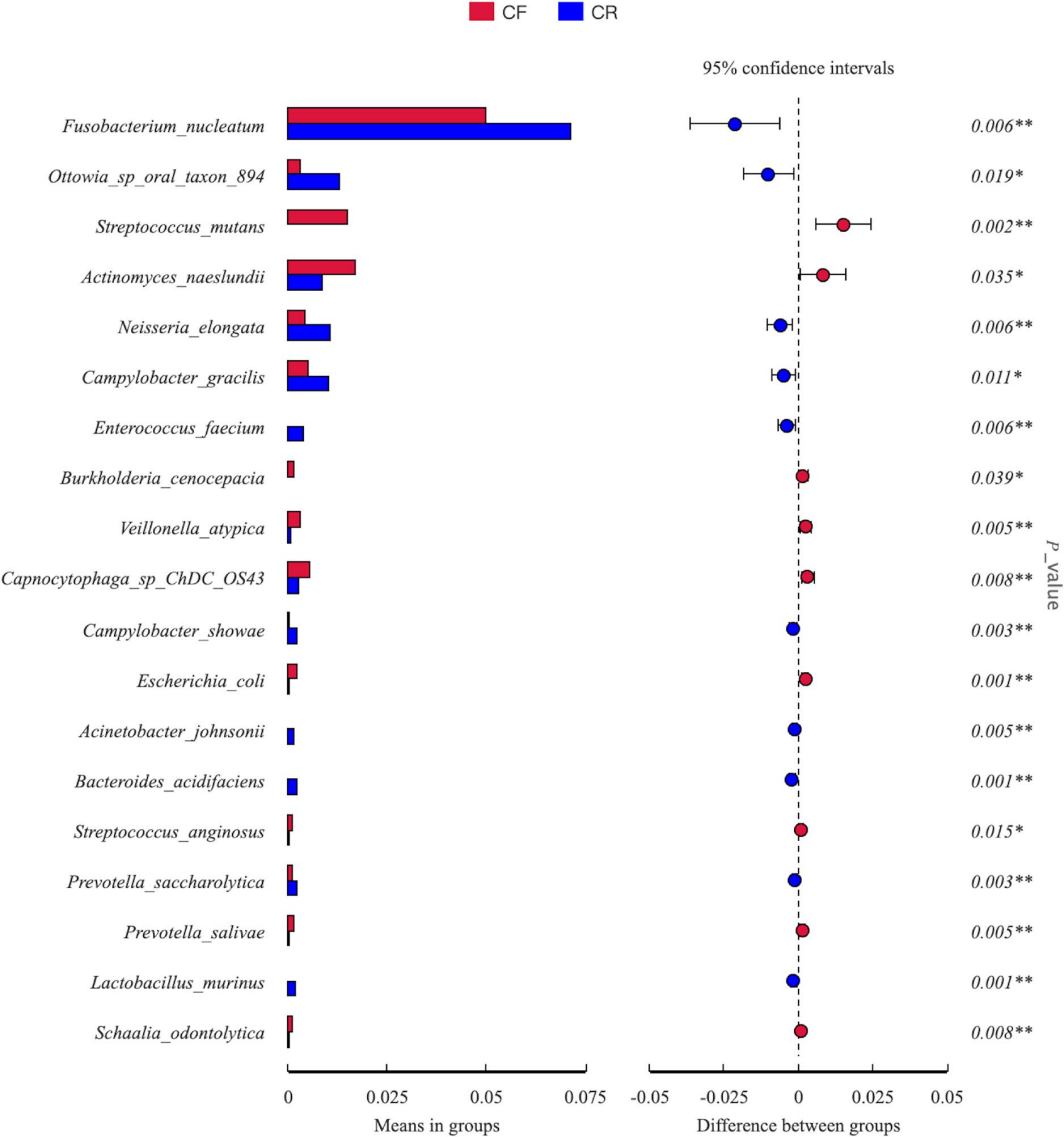
Inter-group comparison of microbial composition at the species level between caries-free (CF) children and children affected by rampant caries (CR)

The inter-group comparison indicated that microbial composition differed between children in the CF and CR groups (Figs. 3, 4, and 5). Anoism test yielded an *R* value of 0.14 and *p* value of 0.001, indicating that there were significant differences in microbial patterns between the CF and CR groups. At the phylum level, when compared to children in the CF group, a lower abundance of *Firmicutes* was detected among children in the CR group (0.16 ± 0.06 vs. 0.22 ± 0.09, *p* = 0.002), while a higher abundance of *Proteobacteria* was detected in the CR group than the CF group (0.20 ± 0.11 vs. 0.13 ± 0.08, *p* = 0.001). *Campylobacterota* was also more abundant among children with rampant caries than the caries-free children (0.02 ± 0.02 vs. 0.01 ± 0.01, *p* = 0.001, Fig. 3).

At the genus level, there were 30 genera with significant differences between the two groups (Fig. 4). *Leptotrichia* was the most abundant genus in both groups. However, its abundance was higher among caries-free children than their peers affected by rampant caries (0.27 ± 0.12 vs. 0.22 ± 0.09, *p* = 0.023). Likewise, *Veillonella* showed higher abundance in the CF group than CR group (0.07 ± 0.08 vs. 0.02 ± 0.02, *p* < 0.001). Conversely, when compared to the CF group, children in the CR group presented with higher abundance of *Neisseria* (0.08 ± 0.07 vs. 0.05 ± 0.04, *p* = 0.007) and *Fusobacterium* (0.07 ± 0.04 vs. 0.05 ± 0.04, *p* = 0.003).

At the species level, a total of 19 species with significant differences in the CF and CR groups were identified. When compared to the CF group, *Fusobacterium nucleatum* (*p* = 0.006), *Ottowia* sp. oral taxon 894 (*p* = 0.019), *Neisseria elongata* (*p* = 0.006), *Campylobacter gracilis* (*p* = 0.011), *Enterococcus faecium* (*p* = 0.006), *Campylobacter showae* (*p* = 0.003), *Acinetobacter johnsonii* (*p* = 0.005), *Bacteroides acidifaciens* (*p* = 0.001), *Prevotella saccharolytica* (*p* = 0.003), and *Lactobacillus murinus* (*p* = 0.001) were more abundant in CR group. Unexpectedly, a lower abundance of *S. mutans* (95%CI 0.01 to 0.02, *p* = 0.002) was detected among children affected by rampant caries, when compared to their peers without caries (Fig. 5).

**Fig. 6:**
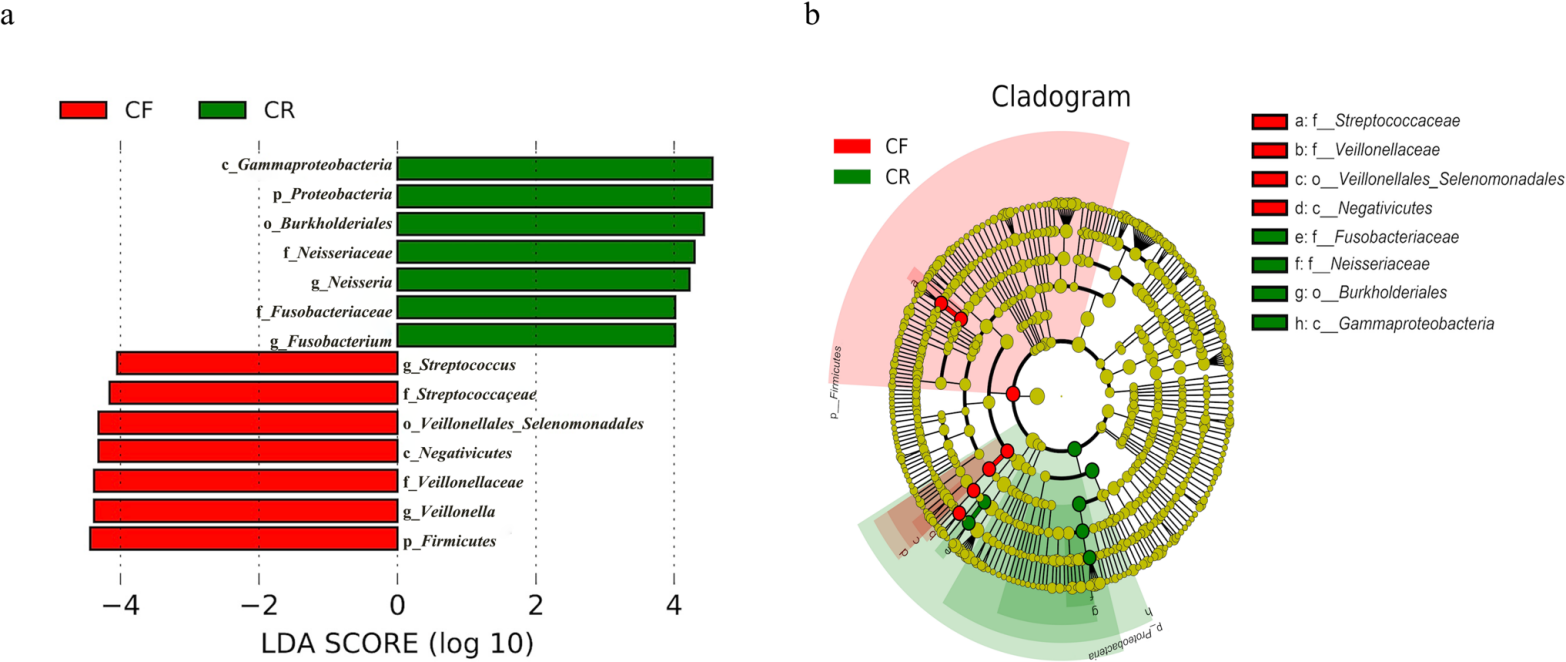
LEfSe analysis between caries-free (CF) children and children affected by rampant caries (CR). **a** Histogram depicting the distribution of LDA scores for abundant features in CF and CR groups. Default LDA score was 4. The length represents the LDA scores. The taxa that were significantly different between the two groups were distinguished by two different colors. **b** Cladogram presenting the taxonomic differences in CF and CR groups, with colored nodes indicating taxa from phyla to the genus level arranged in circles from inner to outer

Additionally, LEfSe analysis indicated that there were four genera with significant differences between the CF and CR groups (Fig. 6a–b). *Veillonella* and *Streptococcus* were enriched among caries-free children, while *Neisseria* and *Fusobacterium* were enriched among children with rampant caries. These results revealed that these taxa may be potential indicators of rampant caries.

**Fig. 7:**
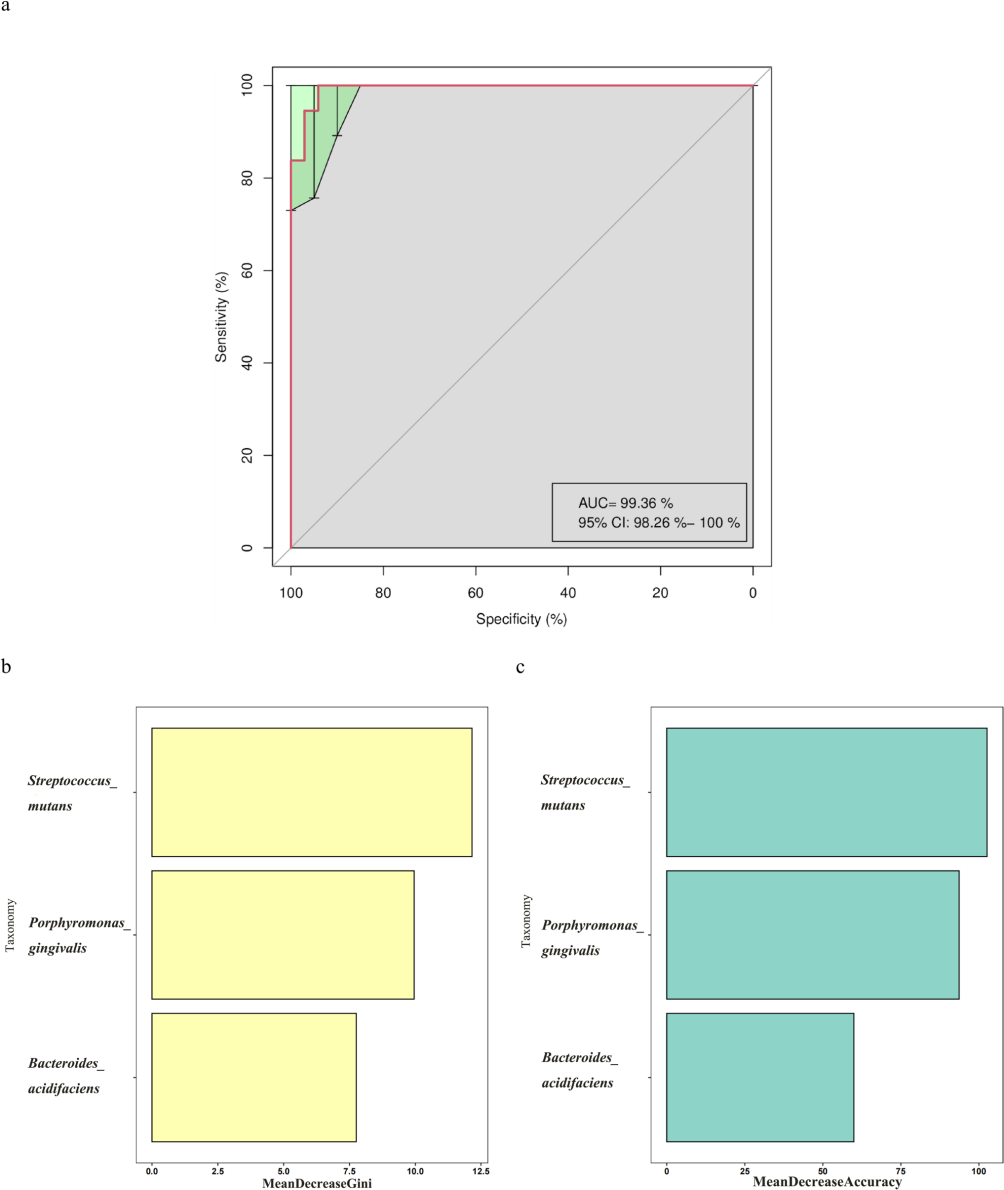
The random forest model constructed for the species taxonomic level to identify biomarkers for children with rampant caries (CR) and caries-free (CF). **a** The ROC of random forest model constructed by 3 species. The AUC: 99.36% (95%CI: 98.26%−100%). The top 3 species were screened by **b** MeanDecreaseAccuracy and **c** MeanDecreaseGini. MeanDecreaseGini evaluates the contribution of a feature to the model’s predictive power by observing the decrease in Gini impurity when that feature is used to split the data; MeanDecreaseAccuracy measures the decrease in model accuracy when a specific feature is excluded from the model. This indicates how essential that feature is for making accurate predictions. AUC: area under the curve; CI: confidence interval; ROC: receiver operating characteristic curve

Moreover, the random forest model indicated that the combination of the three species could differentiate children with rampant caries from children without caries, achieving an area under the receiver operating characteristic (ROC) curve (AUC) of 99.36% (95% CI: 98.26–100%, Fig. 7a). In descending order, the top three most important species were *S. mutans*, *Porphyromonas gingivalis*, and *Bacteroides acidifaciens* (Fig. 7b–c).

### Factors associated with bacterial community structures

**Fig. 8:**
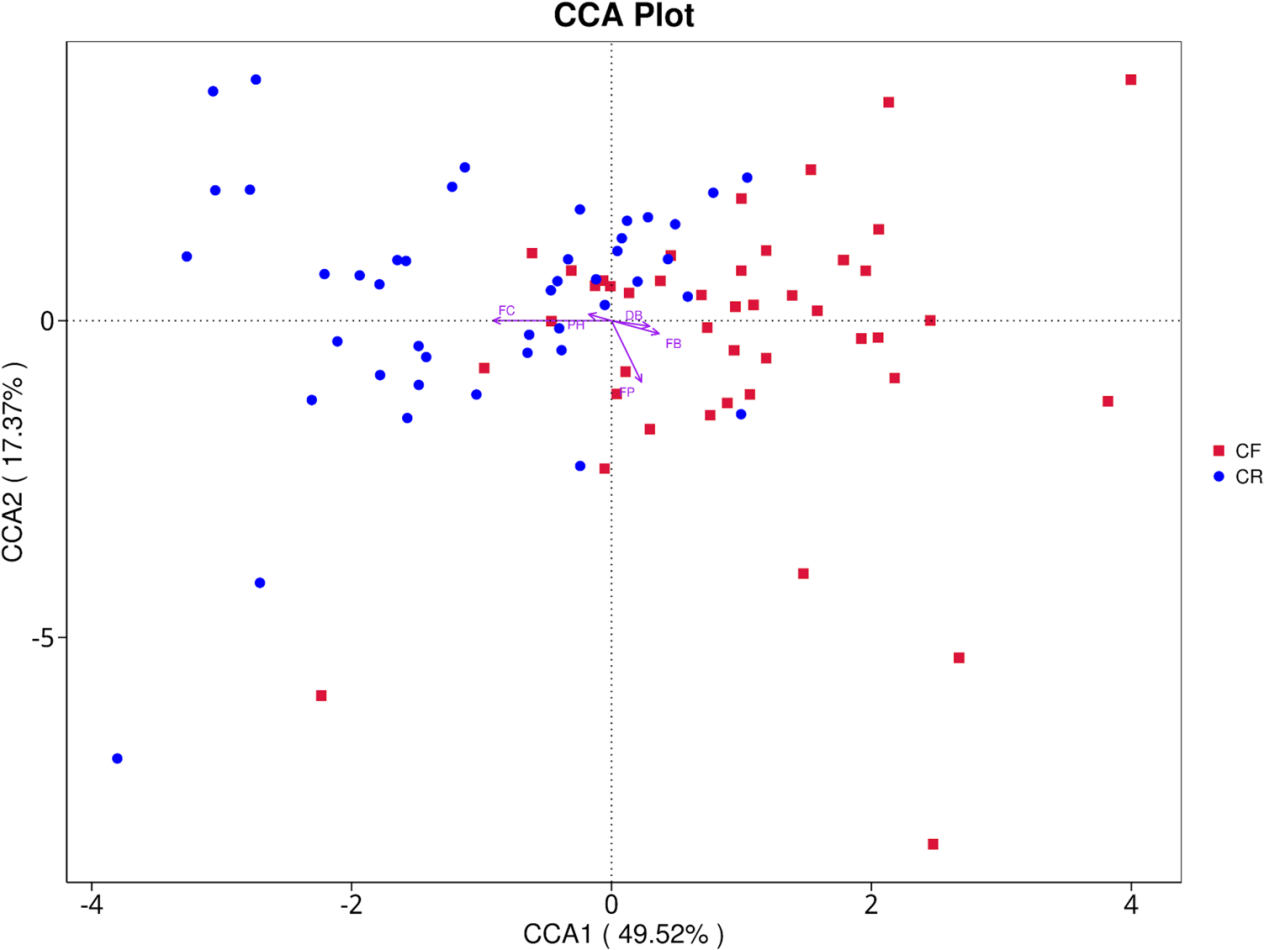
Canonical correspondence analysis (CCA) ordination of environmental factors in caries-free (CF) children and children affected by rampant caries (CR). Each environmental factor is presented by an arrow, with the length of the arrow indicating the correlation. The longer the arrow, the greater the correlation. An acute angle between two factors reveals a positive correlation, while an obtuse angle indicates a negative correlation. Note: FC, frequency of candy consumption; PH, salivary pH level; FP, fluoride toothpaste; FB, toothbrushing frequency; DB, toothbrushing duration

The CCA plot indicated that the bacterial community distribution was mainly influenced by the following factors: frequency of candy consumption (*p* < 0.001), utilization of fluoride toothpaste (*p* < 0.001), and toothbrushing frequency (*p* = 0.05). Additionally, a negative correlation was detected between the frequency of candy consumption and utilization of fluoride toothpaste, and a positive correlation was observed between the toothbrushing frequency and the usage of fluoride toothpaste (Fig. 8).

**Fig. 9:**
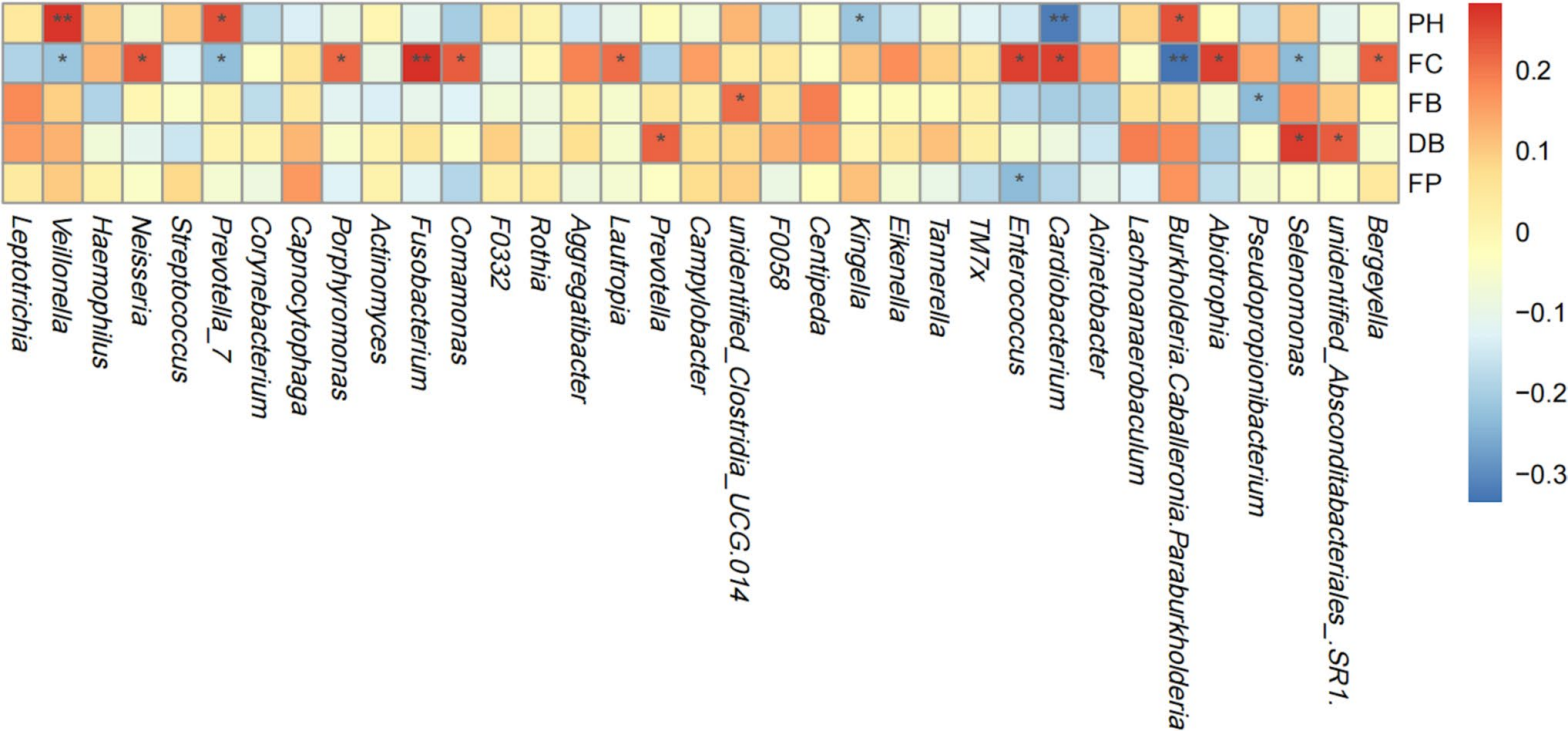
Correlation analysis between genera and potential factors. Positive correlations are represented by red, while negative correlations are represented by blue. The number indicates the Spearman correlation coefficient (*p 0.05; **p < 0.01). Note: PH, salivary pH level; FC, frequency of candy consumption; FP, fluoride toothpaste; FB, toothbrushing frequency; DB, toothbrushing duration

Spearman correlation analysis demonstrated that *Veillonella*, which was enriched among caries-free children, positively correlated with the salivary pH levels (*p* < 0.01), and negatively correlated with the frequency of candy consumption (*p* < 0.05). On the other hand, *Fusobacterium* (*p* < 0.01) and *Neisseria* (*p* < 0.05), which were the taxa that exhibited an increment in abundance among children with rampant caries, displayed a positive correlation with the frequency of candy consumption (Fig. 9).

## Discussion

We conducted a matched case–control study involving 88 children aged 2 to 5 years with primary dentition. Although several studies have explored the variations in bacterial communities between children with and without caries, few studies have focused on preschool children suffering from rampant caries, a condition characterized by its sudden onset, acute progression, and early pulp involvement (Chandra et al. 2007; Namita 2012) . To address this research gap, our case group included children with rampant caries. The severity of caries status is much higher in our study than in prior studies, with nearly 30 affected tooth surfaces per child in the case group, allowing us to investigate the oral microbiome in children with extensive caries. Furthermore, to mitigate potential confounding factors, the gender and age of children in the case and control groups were matched. While the association between salivary pH levels, oral health–related behaviors, and children’s oral health status has been well-documented in epidemiological studies (Ravikumar et al. 2023; Zhou et al. 2019, 2020), the relationship between these factors and the oral microbiome among preschool children remains rarely explored. Our study, therefore, not only compared the composition of oral microbiota between caries-free preschool children and those with rampant caries, but also assessed the impact of salivary pH levels, tooth-brushing habits, and dietary habits on the variation of microbial communities among those children. To our knowledge, this study is the first matched case–control study to examine the oral microbiota profile and its associated factors in preschool children with rampant caries.

Regarding the diversity of the oral microbiota, no significant difference in the alpha diversity indices was detected between the case and control groups. Similarly, another study conducted on Middle Eastern children also observed no difference in alpha diversities among children with and without dental caries (Qudeimat et al. 2021). In contrast, a case–control study conducted by Li et al. (2023) demonstrated greater alpha diversity indices were observed among children with severe early childhood caries than their peers without caries. Moreover, the PCoA and NMDS analysis indicated that the bacterial community structures differed here, suggesting a potentially unique and stable microbial community in children with rampant caries. Peer studies further revealed that when compared to the control group, the caries group exhibited significantly lower diversity (Lee et al. 2016). The lower diversity among individuals with caries was partly due to the elimination of acid-sensitive species within the acidic environment generated by acidogenic bacteria, resulting in the survival of only a few aciduric species (Lee et al. 2016; Rusu et al. 2022). This aligned with our findings that lower salivary pH values were observed among children affected by rampant caries than their caries-free counterparts (*p* = 0.002). In a systematic review, several studies also supported children affected by caries demonstrated lower pH values than their caries-free peers, indicating that salivary pH levels and its buffering capacity contributed significantly to the occurrence of tooth decay (Ravikumar et al. 2023).

This study also revealed that *Leptotrichia*, *Veillonella*, *Neisseria*, *Streptococcus*, and *Prevotella_7* were among the top 10 genera among children in both groups. Among those, *Veillonella* was more abundant in caries-free children. It correlated positively with the salivary pH levels, and negatively with candy consumption frequency. This suggested that *Veillonella* might have a protective effect against caries development. Traditionally, *Veillonella* has been considered to reduce the susceptibility to caries by utilizing the lactic acid produced by other cariogenic bacteria, leading to an increased pH level in the oral cavity (Liu et al. 2020). This was consistent with our findings. However, several studies suggested that *Veillonella* was more common among individuals with active caries, and may contribute to caries progression (Liu et al. 2020; Qudeimat et al. 2021; Tang et al. 2024). In contrast to *Veillonella*, which was found in higher abundance in children without caries, *Neisseria* and *Fusobacterium* genera were more enriched in children with rampant caries, and they showed a positive correlation with the sugary snacking frequency. Hurley et al. (2019) also suggested that *Neisseria* was the most abundant bacterial genus in saliva and dental plaque samples collected from children with severe early childhood caries. Furthermore, they found that *Neisseria* was also a dominant bacterium present in deep dentin lesions of these children. *Neisseria* was reported to be capable of metabolizing glucose, producing organic acids, utilizing lactic acid, reducing the pH in the oral cavity, and increasing the risk of dentin demineralization (Erwin and Gotschlich 1993; Gross et al. 2010). *Fusobacterium* is a primary periodontal pathogen; however, a few studies have also identified its enrichment and predictive potential in relation to caries development (Socransky et al. 1998; Zhu et al. 2018). Chumponsuk et al. (2021) reported a positive correlation between the consumption of carbonated beverages and the abundance of *Fusobacterium*. Similarly, the positive correlation between *Fusobacterium* and sugary snacking consumption was also found in our study. Another study (Fan et al. 2024) found that the abundance of *Leptotrichia* and *Campylobacter* were lower in individuals who frequently consumed high-sugar beverages. In our study, we noted that *Leptotrichia* and *Campylobacter* was less common to be detected among children with frequent sugar intake. However, the differences were not statistically significant (*p* > 0.05). Sugar-rich foods might create an environment conducive to the growth of cariogenic bacteria, potentially leading to an increased prevalence of dental caries (Li et al. 2022). Furthermore, we found that the abundance of *Fusobacterium* was higher among children affected by rampant caries than caries-free children. There is also evidence that the relative abundance of *Fusobacterium* significantly differs between patients experiencing caries recurrence and those without recurrence (Zhu et al. 2018), indicating that *Fusobacterium* might contribute to the occurrence of dental caries.

The random forest model, a machine learning algorithm, has been employed in identifying microbiomes and building predictive models (Blanchet et al. 2020). By using this model, three markers, including *P*. *gingivalis*, *B*. *acidifaciens*, and *S. mutans*, with an AUC of 99.36%, were found to be effective in differentiating preschoolers with rampant caries from their peers without caries. In our study, *P. gingivalis* and *B. acidogenes* were not the top 10 dominant species in the recruited children, with less than 1% abundance in both groups. However, the “keystone” pathogen theory suggests that even species in low-abundance can significantly influence the microbial community, and low-abundance species in the oral microbiome could potentially serve as effective markers for caries (Hajishengallis et al. 2012; Hurley et al. 2019). *S. mutans*, known for its high acidogenic and aciduric properties, is a well-recognized microbiological risk marker of dental caries (Aksakal et al. 2024; Lemos et al. 2019; Wu et al. 2022; Eşian et al. 2017). However, high levels of *S. mutans* do not always correlate with the occurrence of dental caries, and it is not the predominant species in patients with severe caries (Belda-Ferre et al. 2012; Chen et al. 2021; Köhler and Bjarnason 1992; Xiao et al. 2016). In contrast, caries-free individuals have been found to have an enrichment of *S. mutans* (Tu et al. 2022), which aligns with our results.

Although *S. mutans* is a well-identified marker for caries prediction, the roles of *P. gingivalis* and *B. acidifaciens* in caries progression have been rarely reported in previous studies. *P. gingivalis*, widely recognized as a pathogen in periodontal disease, has been linked to pulpal infection or pulp necrosis in primary teeth (Yang et al. 2010; Ito et al. 2011; Lemos et al. 2020). Fabris et al. (2014) collected necrotic pulp and fistula samples from 110 children aged 2 to 12 years. Their findings revealed that *P. gingivalis* was prevalent in necrotic pulp from deciduous teeth in boys from 2 to 5 years old. Given that early pulp involvement is a distinctive characteristic observed in children with rampant caries (Chandra et al. 2007; Namita 2012), it is plausible that *P. gingivalis* can serve as a marker for rampant caries prediction among preschool children. *B. acidogenes*, also identified as a marker in our study, is an adaptable, anaerobic, non-spore-forming, gram-negative, acid-resistant organism that often detected in the gut (Shin et al. 2024). There is evidence that *B. acidifaciens* has the potential to control weight and improve insulin levels (Yang et al. 2017). A study conducted by Then et al. (2020) investigated the impact of diet patterns on gut microbiota in immunodeficient mice transplanted with human cancer. They found a higher abundance of *B. acidifaciens* in mice on a high soluble fiber diet, and it was positively correlated with survival time. Additionally, a recent study (Wu et al. 2024) revealed that patients with ulcerative colitis exhibited a significant reduction of *B. acidogenes* in their feces compared to healthy donors. Similar reductions of *B. acidogenes* were observed in the feces of pigs with lipopolysaccharide-induced intestinal inflammation, indicating the therapeutic potential of *B. acidogenes* in inflammatory intestinal diseases. While these studies demonstrated an essential role of *B. acidogenes* in gut microbiota, the role of *B. acidogenes* in oral microbiota has been underexplored in existing literature. Although our findings suggested that *B. acidogenes* could be a potential marker for rampant caries, further well-designed investigations are necessary to elucidate the mechanisms through which *B. acidogenes* influences rampant caries in pediatric patients.

Additionally, the bivariate analysis of our study revealed that children with a higher frequency of candy consumption had a higher prevalence of caries. Children who did not practice toothbrushing twice daily or who did not use fluoride toothpaste were more likely to be affected by dental caries. Furthermore, lower salivary pH levels were observed in the rampant caries group compared to the control group. These findings are consistent with previous research in this area (Namita 2012; Padmanabha et al. 2017; Wong 2022; Ravikumar et al. 2023). Additionally, we noted that *Enterococcus* tended to exist among children with a higher frequency of candy consumption, while it was less likely to be detected among those children who used fluoridated toothpaste. The growth inhibition effect of fluoride on *Enterococcus* was also supported by Li et al. (2020). These findings indicated that caries prevention interventions could alter the composition of oral microbiome among children with rampant caries.

Despite the aforementioned contributions, this study has several limitations. One limitation is the inconsistent diagnostic criteria for rampant caries in previous studies. Rampant caries is characterized by its acute onset and the presence of advanced or severe decay on multiple tooth surfaces (Winter et al. 1966; Guzmán-Armstrong 2005; Wong 2022). However, the diagnostic criteria vary across previous studies, including (i) carious lesions on the smooth surfaces of at least two upper incisors (Al-Malik et al. 2002); (ii) carious lesions on palatal or buccal or labial surfaces of two or more upper incisors (Winter et al. 1966); (iii) caries affecting at least two maxillary incisor area and an overall dmft score of ≥ 8 (Liu et al.); (iv) at least 6 active carious lesions on primary teeth (Vinckier et al. 2001); (v) “multiple (> 5) cervical, proximal, or lingual white spot or brown spot lesions or shallow active caries lesions in enamel or dentine” (Kuriakose et al. 2013). Rampant caries is characterized by its sudden appearance, acute burrowing, early pulp involvement, and advanced progress with over 10 new lesions appearing each year on tooth surfaces typically less vulnerable to caries (Chandra et al. 2007; Namita 2012). Given that the main characteristic of rampant caries is its sudden onset and rapid progression, we chose to follow the above criteria, which better captures the onset and progression characteristic of this condition. Consequently, the children in our case group presented with severe caries status. Previous studies have delved into alterations in oral microbiota between children with and without ECC. Our stringent inclusion criteria allow us to compare bacterial communities between children with more severe caries statuses and their caries-free counterparts. This approach contributes to the novelty of our study. Furthermore, Wu et al. (2022) revealed that, beyond bacteria, fungi, particularly *Candida albicans*, might contribute to the development of dental caries. Another study conducted by Fechney et al. (2018) employed Illumina MiSeq to sequence the ITS2 region, revealing that three species of fungi were enriched in children with caries. Interestingly, they found *C. albicans* might not contribute to caries development as previously assumed. However, in our study, we used the 16S rRNA sequencing technique to analyze the oral microbiota of the recruited children. This method, while effective for bacterial analysis, does not provide information on fungal composition. Therefore, the role of fungi was not examined in our study. For future studies, we recommend applying comprehensive analysis techniques, which can assess bacterial, fungal, or even viral compositions. This would offer a more holistic view of the role of oral microbiota in rampant caries, potentially paving the way for more effective preventative measures and treatments.

Within the limitation of this study, we observed alterations in the oral microbiota composition, salivary pH levels, and oral health–related behaviors between preschool children with rampant caries and their counterparts without caries. Children’s oral microbiota was influenced by factors such as frequency of candy consumption, utilization of fluoride toothpaste, and toothbrushing frequency. *P. gingivalis*, *B. acidifaciens*, and *S. mutans *were identified as markers for distinguishing preschoolers with rampant caries from their caries-free peers. While *S. mutans* is a well-identified risk marker for dental caries, the roles of *P. gingivalis* and *B. acidifaciens* in dental caries development are seldom reported. Previous studies have revealed the relationship between *P. gingivalis* and pulp infections, whereas *B. acidifaciens* has been associated with intestinal health conditions. More well-designed cohort studies are recommended to explore the relationship between gut microbiota and the oral health status of pediatric patients, which could potentially provide innovative insights into effective caries management strategies for young children.

## Data Availability

The Illumina sequencing raw data has been deposited in SRA database (Ref No. PRJNA1141721).
